# Comparative Assessment of Lymph Node Micrometastasis in Cervical, Endometrial and Vulvar Cancer: Insights on the Real Time qRT-PCR Approach versus Immunohistochemistry, Employing Dual Molecular Markers

**DOI:** 10.1155/2014/187684

**Published:** 2014-01-02

**Authors:** Kalliopi I. Pappa, Alexandros Rodolakis, Ioanna Christodoulou, Maria Gazouli, Sofia Markaki, Aris Antsaklis, Nicholas P. Anagnou

**Affiliations:** ^1^First Department of Obstetrics and Gynecology, University of Athens School of Medicine, Alexandra Hospital, 11528 Athens, Greece; ^2^Laboratory of Biology, Department of Basic Medical Sciences, University of Athens School of Medicine, 11527 Athens, Greece; ^3^Laboratory of Cell and Gene Therapy, Center of Basic Research II, Biomedical Research Foundation of the Academy of Athens, 11527 Athens, Greece; ^4^Department of Pathology, Alexandra Hospital, 11528 Athens, Greece

## Abstract

To address the value of qRT-PCR and IHC in accurately detecting lymph node micrometastasis in gynecological cancer, we performed a systematic approach, using a set of dual molecular tumor-specific markers such as cytokeratin 19 (CK19) and carbonic anhydrase 9 (CA9), in a series of 46 patients (19 with cervical cancer, 18 with endometrial cancer, and 9 with vulvar cancer). A total of 1281 lymph nodes were analyzed and 28 were found positive by histopathology. Following this documentation, 82 lymph nodes, 11 positive and 71 negative, were randomly selected and further analyzed both by IHC and qRT-PCR for CK19 and CA9 expression. All 11 (100%) expressed CK19 by IHC, while only 6 (54.5%) expressed CA9. On the contrary, all the histologically negative for micrometastases lymph nodes were also negative by IHC analysis for both markers. The comparative diagnostic efficacy of the two markers using qRT-PCR, however, disclosed that the analysis of the same aliquots of the 82 lymph nodes led to 100% specificity for the CK19 biomarker, while, in contrast, CA9 failed to recapitulate a similar pattern. These data suggest that qRT-PCR exhibits a better diagnostic accuracy compared to IHC, while CK19 displays a consistent pattern of detection compared to CA9.

## 1. Introduction

Currently, a considerable percentage of women with gynecological cancer associated with histologically negative lymph nodes develop relapse. This important clinical issue has led to further investigations on the putative factors that can lead to this particular biological behavior [1]. Several studies on the evaluation of patients with melanoma [2] or breast cancer [3] suggested as a potential cause of relapse, the presence of micrometastasis, defined as tumor deposits measuring 0.2–2 mm in apparently negative lymph nodes [4]. Thus, the practice of lymphadenectomy has eventually emerged and still remains the crucial standard of staging of gynecological cancer, as applied to cervical, endometrial, and vulvar cancer. Its importance at the time of surgery is underscored by the significant effects on the five-year survival rates. Further technical advancements employing several aspects of laparoscopy have significantly improved its utility and resulted in the development of new techniques, such as laparoscopic-assisted radical vaginal hysterectomy [5] and radical vaginal trachelectomy [6].

Although lymph node metastasis is considered nowadays an established prognostic factor for gynecological cancer, there is still a need for a consensual histologic definition of micrometastasis, which can eventually enable the development of a reliable and reproducible staging system [4]. More specifically, a major current issue towards the development of such standards is the significant variability of the incidence of micrometastasis depending on the evaluation techniques employed [7]. These techniques currently involve (a) conventional staining with hematoxylin-eosin, (b) ultrastaging, that is, further examination of additional wide intervals by hematoxylin-eosin, coupled accordingly with or without immunohistochemical analysis [8], (c) sentinel lymph node biopsy [9, 10], and (d) reverse transcriptase-polymerase chain reaction (RT-PCR) analysis for cytokeratin expression [11–13].

Regarding the technique of the sentinel lymph node biopsy, defined as the analysis of the first lymph node draining a tumor, it initially seemed to have provided an alternative assessment of the lymph node state, avoiding formal lymphadenectomy [7]. Recent studies have provided evidence for the utility of the sentinel lymph node versus the nonsentinel lymph node mapping for endometrial cancer, in improving the detection of metastatic disease in regional lymph nodes [14], although several studies have documented that even when serial sectioning is used, very small clusters of tumor cells can escape immunohistochemistry (IHC) staining [15, 16]. Nevertheless, the assessment of the lymphatic spread in gynecological cancer involving either lymphadenectomy or no nodal dissection or lymphatic mapping using sentinel lymph node still remains controversial, providing no consistent or convincing results [17–19], while it is associated with high false-negative rates up to 50% [20]. Therefore, due to yet unresolved technical, clinical, and safety issues of the sentinel lymph node concept, the biopsy of this particular lymph node alone is currently not a routine procedure for gynecological cancer assessment of micrometastasis [19].

On the contrary, the real time quantitative reverse transcriptase-polymerase chain reaction (qRT-PCR) technology, representing a highly sensitive method for the detection of lymph node micrometastases, by virtue of its capacity to identify single tumor cells of epithelial origin, has been employed so far in a rather limited number of clinical studies, either in vulvar [21], cervical [11, 13, 22, 23], or endometrial [12] cancer, employing established epithelial markers like cytokeratin 19 [11, 13, 22] and cytokeratin 20 [12] or tumor-specific isozyme markers such as carbonic anhydrase 9 [21]. However, due to several limitations of specificity and reproducibility, this approach has not been fully evaluated so far in the molecular quantification and mapping of lymph node micrometastases.

Thus, based on the above limited data, the clinical value of real time qRT-PCR approach in micrometastases detection in gynecological cancer needs to be clarified by additional studies [7]. In view of this incomplete status of the field and in order to address and refine the value of the real time qRT-PCR technique compared to IHC, for eventual detection of micrometastases in routine practice, in the present study, we opted a systematic approach to optimize its specificity and assess its validity in reliably detecting lymph node micrometastases, by using for the first time a set of dual molecular tumor-specific markers such as cytokeratin 19 (CK19) and carbonic anhydrase 9 (CA9) in a series of patients with cervical, endometrial, and vulvar cancer.

## 2. Materials and Methods

### 2.1. Patients

This study, involving three series of consecutive patients with cervical, endometrial, and vulvar cancer, was approved by the Ethics Committee of the Alexandra Hospital in Athens. All patients signed a fully-written informed consent. The study enrolled a total of 46 patients, including 19 patients with cervical cancer, 18 patients with endometrial cancer, and 9 patients with vulvar cancer. Control samples of histologically normal lymph nodes and tissues were obtained from biopsy of the corresponding lymph nodes and tissues, respectively, from patients undergoing surgery for benign gynecological diseases. The tumor samples were classified according to the new 2009 FIGO cancer staging system [24] and the histological classification system of WHO [25]. None of the patients had received any preoperative chemotherapy or irradiation treatment.

### 2.2. Lymph Node Identification

A total of 1281 lymph nodes derived from all cases were identified. The samples were obtained following dissection of either pelvic (iliac and obturator) or inguinal lymph nodes in patients undergoing surgery for either cervical, endometrial, or vulvar cancer at the First Department of Obstetrics and Gynecology of the University of Athens at the Alexandra Hospital in Athens. All surgically removed lymph nodes were initially examined histopathologically on sections of up to 3 *μ*m, using routine hematoxylin and eosin (HE) staining, and independently by two expert histopathologists, without knowledge of the clinicopathologic information or of the CK19 and CA9 levels. Lymph nodes exhibiting metastatic deposits of ≤2 mm within largest dimension were designated to harbor micrometastatic disease, while those containing metastatic deposits of >2 mm were considered to have macrometastatic disease, according to the recommendations of the American Joint Committee for Cancer Staging [4]. Each lymph node was labeled and bisected along the length into two bivalves. One half was snap-frozen in liquid nitrogen and stored at −86°C until extraction of RNA. The other half was fixed in formalin and embedded in paraffin for conventional histopathology with HE and immunohistochemistry (IHC) evaluation employing anti-CK19 and anti-CA9 antibodies. Twenty-eight lymph nodes from the total of 1281 lymph nodes dissected from the series of the 46 patients were found positive for micrometastasis by histopathology. Following this documentation, eighty-two lymph nodes, that is, 11 histopathologically positive and 71 histopathologically negative lymph nodes were randomly selected and were further analyzed both by IHC and qRT-PCR for the expression of CK19 and CA9. Lymph nodes from (a) two cases of cervical cancer, a human colon adenocarcinoma cell line DLD-1 [26], and a human erythroleukemia K562 cell line [27] and (b) a biopsy specimen from a case with renal clear cell carcinoma and from one case of colorectal cancer confirmed by routine pathology were selected as positive control samples for CK19 and CA9 expression, respectively, while three cases of lymph nodes of benign origin were used as negative controls.

### 2.3. Immunohistochemistry (IHC)

Immunohistochemical analysis (IHC) of the 82 bivalve lymph nodes was performed in the fully automated IHC and ISH Leica Bond-Max system (Leica, Wetzlar, Germany). Briefly, the bivalves were cut into serial 3 *μ*m lymph node sections at 10 *μ*m intervals and were automatically deparaffinized employing the Bond Dewax Solution and the Novocastra Epitope Retrieval Solutions 1 (pH 6) and 2 (pH 8). This was followed by (a) antibody labelling, employing the Bond Polymer Refine Red Detection Kit—a biotin-free system avoiding nonspecific background staining due to endogenous biotin—and (b) conjugation of the antibody with polymeric alkaline phosphatase. This approach, utilising the Fast Red Chromogen, permits the visualisation of the complex as a red precipitate. Finally, counterstaining of the sections was performed with hematoxylin. For the detection of the CK19 expression, a mouse monoclonal anti-human antibody (Clone RCK 108) was used (Dako Denmark A/S, Glostrup, Denmark). Similarly, for the CA9 expression, a rabbit polyclonal antibody (ab 15086) was used (Abcam Plc., Cambridge, UK). All prepared slides were labelled using randomised numbering, and were evaluated independently by two expert pathologists without knowledge of the clinicopathologic information.

### 2.4. RNA Preparation

Total cellular RNA from the snap-frozen samples was prepared using TRIzol (Invitrogen by Life Technologies, Carlsbad, CA, USA) and was further purified employing PCI (phenol : chloroform : isoamyl alcohol; 25 : 24 : 1) extraction [28] and was assayed using the Genequant II DNA/RNA Calculator spectrophotometer (Pharmacia, Uppsala, Sweden).

### 2.5. Real Time qRT-PCR Assay for Cytokeratin 19 (CK19) and Carbonic Anhydrase 9 (CA9)

Reverse transcription was performed employing the QuantiTect Reverse Transcription Kit from Qiagen (Hilden, Germany) according to the manufacturer's instructions. Specifically, 1 *μ*g of total RNA was pre-incubated in gDNA Wipeout Buffer, 7x and RNase-free water at 42°C for 2 min. Following genomic DNA elimination, the RNA samples were reverse transcribed using a master mix prepared from Quantiscript Reverse Transcriptase, Quantiscript RT Buffer, and RT Primer Mix at 42°C for 15 min and then inactivated at 95°C for 3 min. Following this step, real time qRT-PCR assays were performed on a Roche LightCycler 2.0 detection system (Roche Diagnostics GmbH, Mannheim, Germany) in 20 *μ*L volumes in glass capillaries, employing the QuantiTect Probe RT-PCR Kit from Qiagen (Hilden, Germany) according to the manufacturer's instructions. Briefly, an initial incubation step of 15 min at 95°C was performed for HotStarTaq DNA Polymerase activation, followed by 45 cycles of PCR with denaturation at 95°C for 30 sec; annealing at 56°C for 40 sec; extension at 72°C for 40 sec; and a single final step at 72°C for 5 min, in the presence of 1x QuantiTect Probe PCR Master Mix buffer, 0.5 *μ*M primers, 0.2 *μ*M probe, 0.5 units of uracil-N-glycosylase (UNG) and ≤1 *μ*g of cDNA. Reactions were performed in duplicate. Post-amplification denaturation curves showed that the primer pairs generated single products. Data were analysed using the comparative CT method for the relative quantitation of results.

For the real time qRT-PCR assays, the following sets of primers and probes were employed. Namely, a specific set of CK19 (GenBank accession number Y00503) primers (forward 5′-TCG ACA ACG CCC GTC TG-3′; reverse 5′-CCA CGC TCA TGC GCA G-3′) and probe (6FAM-CCG AAC CAA GTT TGA GAC GGA ACA GG-TMR) were designed using the Primer Express software v1.6 (Applied Biosystems, Foster City, CA, USA) and obtained from VBC-Biotech GmbH (http://www.vbc-biotech.at/). The selection of these primers spanning exons 2 and 3, respectively, avoids the amplification of the CK19**α** pseudogene (GenBank accession number M33101), since the probe and the reverse primer contain three and two mismatches, respectively [13]. Additionally, this strategy [13] avoids the amplification of the CK19*b* pseudogene (GenBank accession number U85961) due to several mismatches of the primers and a deleted region of the pseudogene, complementary to the probe. Similarly, a specific set of CA9 (GenBank accession number NM001216) primers spanning exons 9 and 11, respectively (forward 5′-GCT GCT GAG CCA GTC CA-3′; reverse 5′-GGC GGT AGC TCA CAC CC-3′) and probe (6FAM-CTG CCT TCT CAT CTG CAC AAG GAA C-TMR) were designed and obtained from TIB MOLBIOL Syntheselabor GmbH (Berlin, Germany; http://www.tib-molbiol.com/).

Normalization of the expression levels of cytokeratin 19 (CK19) and carbonic anhydrase 9 (CA9) mRNAs was performed using as a reference the glyceraldehyde 3-phosphate dehydrogenase (GAPDH) gene mRNA (GenBank accession number G04038), which is constitutively expressed at high levels in most tissues and cells, exhibiting no variability or tissue specificity. This normalization approach removes inaccuracies due to variations in reverse transcription efficiency, since the reference gene mRNA is reversibly transcribed along with the CK19 and CA9 mRNAs. The specific set of GAPDH primers (forward 5′-GAA GAT GGT GAT GGG ATT TC-3′; reverse 5′-GAA GGT GAA GGT CGG AGT C-3′) and probe (6FAM-CAA GCT TCC CGT TCT CAG CC-TMR) were also obtained from VBC-Biotech GmbH.

## 3. Results

### 3.1. Clinicopathological Data

The clinical parameters and the histopathological features of the 46 patients with cervical, endometrial, and vulvar cancer are summarised in Table 1. Cervical cancer was predominantly (73.6%) represented as keratinizing squamous cell carcinoma, while the majority (84.2%) of the patients were at stage I. The group of endometrial carcinoma consisted mostly (61.1%) of endometrioid adenocarcinoma, assessed primarily (77.7%) at stage I. Finally, vulvar carcinoma patients exhibited almost exclusively (88.8%) a keratinizing squamous cell carcinoma histology, associated with a variant tumor stage.

### 3.2. Lymph Node Metastases Detected by Conventional Histopathology Techniques

The majority of patients (70%) underwent radical pelvic and/or inguinal lymph node resection, that is, >20 lymph nodes, as shown in Table 1, while the median number of resected pelvic and/or inguinal lymph nodes was 28 (range 3 to 60 lymph nodes). A total of 1281 lymph nodes were obtained from the cohort of the 46 patients who underwent intraoperative evaluation of lymph nodes as described in Section 2. Lymph node micrometastases were detected in 28 lymph nodes derived from 11 patients out of the total cohort of 46 patients (23.9%) employing routine histologic examination.

### 3.3. CK19 and CA9 Expression in Lymph Nodes Detected by IHC Analysis

**Figure 1:**
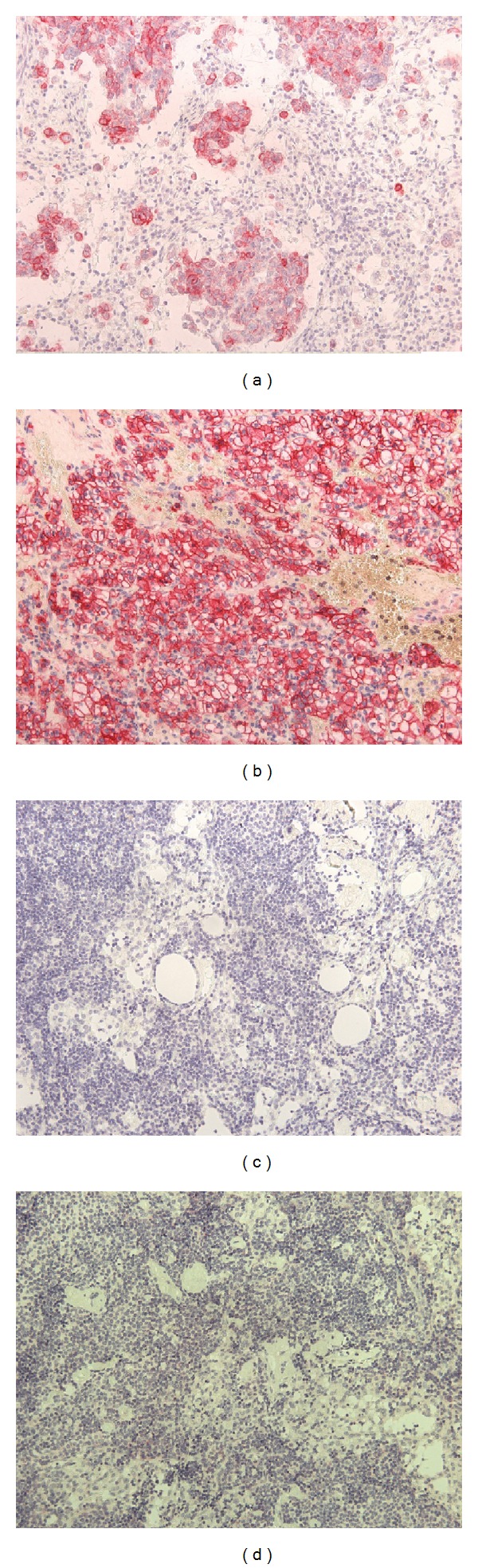
Immunohistochemical analysis of positive and negative control samples for CK19 and CA9 expression. (a) Lymph node positive for metastasis from a patient with cervical carcinoma, stained with the mouse anti-CK19 monoclonal antibody. (b) A case of clear cell renal carcinoma positive for expression of CA9, using the rabbit anti-CA9 polyclonal antibody. (c) Lymph node from patient IE-19 with endometrial carcinoma, negative for micrometastasis and stained with the anti-CK19 antibody. (d) Lymph node from patient IE-19 with endometrial carcinoma, negative for micrometastasis and stained with the anti-CA9 antibody.

**Figure 2:**
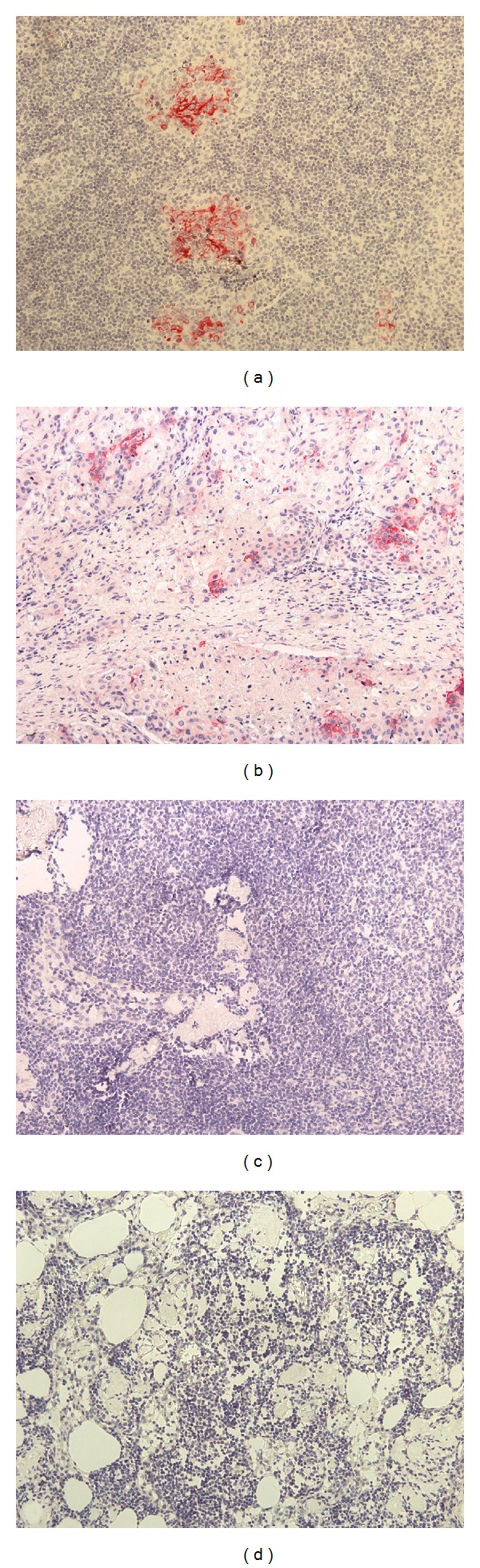
Immunohistochemical assessment for micrometastasis of representative lymph nodes by CK19 and CA9 expression. (a) Lymph node from patient IE-12 with endometrial carcinoma, positive for micrometastasis, exhibiting CK19 expression by the clusters of metastatic epithelial cells. (b) Lymph node from patient IC-8 with cervical carcinoma, positive for micrometastasis, exhibiting CA9 expression by the isolated metastatic epithelial cells. (c) Lymph node from patient IE-12 with endometrial carcinoma, negative for micrometastasis, stained with the anti-CK19 antibody. (d) Lymph node from patient IC-23 with cervical carcinoma, negative for micrometastasis and stained with the anti-CA9 antibody.

Following the initial evaluation of all samples employing routine HE staining, a total of 82 lymph nodes, representing histopathologically 11 positive and 71 negative lymph nodes, were prepared for further IHC analysis. All positive control samples for either CK19 or CA9 proteins, demonstrated positive staining by IHC analysis, respectively (Figures 1(a) and 1(b)), while all negative control samples exhibited lack of expression of the two markers (Figures 1(c) and 1(d)). Furthermore, when the 11 positively detected lymph nodes by conventional histopathologic techniques were immunostained with anti-CK19 or anti-CA9 antibody, all 11 (100%) expressed the CK19 protein (Figure 2(a) and Table 2), while only 6 (54.5%) expressed the CA9 protein (Figure 2(b) and Table 2). Interestingly, three of these six lymph nodes, derived from three patients designated as IC-8, IV-5 and IV-6 (Table 1), were positive for both C19 and CA9 expression. On the contrary, all the histologically negative for micrometastases lymph nodes, were also negative by IHC analysis for both proteins (Figures 2(c) and 2(d) and Table 2).

### 3.4. CK19 and CA9 Expression in Lymph Nodes Detected by Real Time qRT-PCR

Real time quantitative RT-PCR (qRT-PCR) analysis was used to assess the selected 82 lymph nodes derived from 46 patients enrolled in this study, for CK19 and CA9 RNA expression. The qRT-PCR assay disclosed that all positive clinical control specimens and those of the human colorectal cell line DLD-1 [26] and the human erythroleukemia K562 cell line [27], were displaying either CK19 or/and CA9 expression, while all negative controls exhibited absence of their expression. At least one of the tested lymph nodes from each of the 35 patients who were negative for metastases by conventional histopathologic technique, was found also negative for CK19 and CA9 by qRT-PCR assay. All 11 lymph nodes which were positive for CK19 by IHC analysis, were found to express CK19 by qRT-PCR assay. Furthermore, the qRT-PCR assay for CK19 expression was slightly more sensitive than conventional histopathologic analysis and IHC for micrometastases, by detecting 2 additional positive lymph nodes from the total of 71 lymph nodes which were found to be negative for CK19 by IHC analysis (Table 2). Concerning the CA9 expression, all 6 lymph nodes detected being positive for micrometastases by IHC, were found also to express CA9 by qRT-PCR assay, while an additional lymph node of the total 76 lymph nodes found to be negative by IHC, was documented as positive for CA9 expression by qRT-PCR (Table 2).

## 4. Discussion

In the present study, we performed a comprehensive analysis for the assessment of the specificity and validity of the two major approaches, that is, IHC and qRT-PCR, currently utilised for the detection of micrometastases in gynecological cancer. More specifically, we wished to directly assess the diagnostic efficacy of the two methods in detecting micrometastases in the lymph nodes of a series of 46 patients with gynecological cancer, involving either cervical, endometrial, or vulvar carcinomas. Our approach was based on the simultaneous evaluation of the diagnostic capacity and sensitivity of specific epithelial markers, such as CK19 and CA9, which are constitutively expressed in cervical, endometrial and vulvar cancer, but not in normal lymph nodes [29]. Thus, the expression of such relatively specific markers, provides the opportunity to accurately detect cancer cells that metastasize into lymph nodes. Expression of CK19 has been used so far as a single marker for the detection of micrometastases in gynecological cancer [11, 13, 22], while a single study has employed another member of the cytokeratin family, the CK20 protein [12]. Similarly, a single study on the micrometastasis of vulvar carcinoma [21], has utilised a tumor-specific isomarker, such as CA9. This approach has been successful in other forms of cancers [30]. Furthermore, besides cervical epithelium, CA9 exhibits a unique feature of being expressed in several types of tumors, such as renal carcinoma [31], but in contrast to the pattern of CK19 expression, CA9 is not expressed in the corresponding normal tissues of these types of cancer. Therefore, we chose this additional isoform as a cancer-specific marker for our study.

The above studies have utilised either a series of patients with a single type of gynecological cancer [11–13, 21–23] or a single metastasis-specific marker [11–13, 21, 22], making the assessment of the individual role of these markers and methodologies rather inconclusive. To address these technical difficulties, and to independently evaluate the contribution of each parameter, in the presented study, we opted to (a) cover the entire spectrum of gynecological cancer, by including patients with cervical, endometrial and vulvar cancer, primarily with early stages of cancer and (b) utilise simultaneously two biological epithelial markers with distinct features, one (CK19) being expressed both in normal and in gynecological cancer and one (CA9) lacking expression in normal cervical, endometrial, and vulvar epithelium, but being ectopically expressed in gynecological cancer.

Our data on the analysis of a total of 1281 lymph nodes derived from a series of 46 patients, provide several new features of potential importance. First, they demonstrate that the approach of qRT-PCR overall has a better diagnostic accuracy in detecting micrometastasis compared to IHC. All 11 tested lymph nodes that were found histopathologically positive by the initial conventional staining of HE exhibited CK19 expression by qRT-PCR, while 2 additional lymph nodes of the initial 71 negative ones by HE and IHC showed also CK19 expression by qRT-PCR. On the contrary, only 6 of the 11 positive lymph nodes by HE staining, were detected positive for CA9 by IHC.

These data on CK19 expression employing either IHC or qRT-PCR methodologies are consistent with previous studies for micrometastasis in cervical [11, 13, 22] or in endometrial carcinoma [12]. Specifically, a specificity ranging from 100% [11] to 80.4% [22] has been documented in lymph nodes of patients with early-stage cervical cancer, while the use of an alternative cytokeratin such as CK20 documented that qRT-PCR of CK20 exhibits higher sensitivity than histopathological methods [12]. Similar to the data of our study, recent results on endometrial cancer micrometastasis [32] document also the very low incidence of the IHC-positive micrometastases in HE-negative lymph nodes, a fact not justifying routine IHC staining, while at the same time they suggest that until now, little evidence exists to support the putative clinical significance of IHC-stained micrometastases in endometrial cancer. Finally, cytokeratin staining performed with AE1/AE3 antibodies in a series of lymph nodes from patients with endometrial cancer has documented the improvement of the sensitivity for the detection of metastases compared to HE [33].

Second, our data provide novel features on the comparative diagnostic efficacy of the second tested tumor-specific marker of CA9. Our data disclosed that the analysis of the same aliquots of the 82 selected lymph nodes initially characterised by HE led to 100% specificity by employing qRT-PCR and the CK19 biomarker, while in contrast, the same methodology utilising the tumor-specific marker CA9 failed to recapitulate a similar pattern of diagnostic accuracy. Apparently, this discrepancy does not reflect putative inherent aspects of the qRT-PCR methodology, since the same approach was applied for the detection of both markers. Thus, this differential activity probably underlies the peculiarities of CA9 expression. This finding is slightly different from the data of a study on the detection of CA9 by RT-PCR, in a series of both inguinal and sentinel lymph nodes derived from patients with vulvar carcinoma, where full correlation between RT-PCR and standard HE staining was noted in 75% of samples [21]. The relatively increased sensitivity of CA9 expression using the RT-PCR in the latter study compared to the present data may be attributable to the fact that in the study of vulvar carcinoma [21], about 50% of the tested lymph nodes were designated as sentinel nodes, representing the first nodes in the lymphatic flow from the primary lesion, and it is expected to be the first site of a metastatic process. Thus, it is conceivable that the analysis of this specific subgroup of lymph nodes provided additional sensitivity.

Compared to CK19, the role of CA9 in carcinogenesis has not been entirely delineated. Its expression is characteristically induced by hypoxia via the hypoxia-inducible factor-1 (HIF-1), a common but not a consistent feature of tumor growth [21]. Since the tissue oxygen content has been shown to be the main regulatory mechanism for CA9 upregulation, a plausible explanation for the discordant and inconsistent expression of CA9 compared to CK19 in the same bivalve derived from the individual patient could be the different degree of hypoxia among the lymph nodes tested. Actually, this notion of heterogeneity is indirectly supported by the fact that three of the six lymph nodes positive for CA9 by IHC were positive for both markers.

In the present work, we did not focus on the evaluation of the sentinel lymph node, since the main aim of the study was the direct assessment of the specificity and sensitivity of the two methods employing dual markers and utilizing lymph nodes derived from full lymphadenectomy. It is conceivable that sentinel lymph node qPCR analysis coupled with IHC using these markers could serve to reveal best the sentinel-specific micrometastasis status, which could eventually lead towards performing less radical surgery [34]. However, this assumption, along with the need for validation of the established differential pattern of expression of the two markers documented in the present study, should be confirmed and further evaluated by a perspective randomized multicenter study, employing an even wider spectrum of gynecological cancer patients. Our group is planning with other centers to conduct such type of trial, which can formulate future strategies for accurate metastasis detection. Similar novel approaches are currently in progress, involving a one-step nucleic acid application (OSNA), which detects metastasis by quantifying the CK19 mRNA levels, irrespectively whether it is expressed or not, by employing direct analysis of the supernatant of the homogenised lymph node prior to mRNA purification and exhibiting also good correlation with the cancer cells of the lymph node [35–38].

## 5. Conclusions

In summary, the data from our studies on the detection of micrometastasis in three types of gynecological cancer employing dual biomarkers clearly suggest that qRT-PCR seems to exhibit a slightly better diagnostic accuracy compared to IHC and corroborate previous studies, while the epithelial marker of CK19 when directly compared to the CA9 tumor-specific marker displays a rather consistent pattern of accurate detection of micrometastasis, which is independent of other variable tumor parameters, such as hypoxia and oxygen levels.

## Figures and Tables

**Table 1:** Clinicopathological data of the series of 46 patients tested for micrometastases.

Patient code	Location	Histological classification	Stage	Grade	Lymph nodes (positive/total)
IC5	Cervix	Squamous cell carcinoma keratinizing	Ib1	1	0/40
IC6	Cervix	Squamous cell carcinoma keratinizing	Ib1	3	0/47
IC8	Cervix	Squamous cell carcinoma keratinizing	Ib2	2	8/48
IC9	Cervix	Squamous cell carcinoma keratinizing	Ib2	1	2/19
IC10	Cervix	Squamous cell carcinoma keratinizing	Ib1	2	0/34
IC11	Cervix	Villoglandular papillary adenocarcinoma	Ib1	2	0/29
IC12	Cervix	Squamous cell carcinoma keratinizing	Ib1	2	0/53
IC14	Cervix	Squamous cell carcinoma keratinizing	Ia1	In situ	0/18
IC15	Cervix	Squamous cell nonkeratinizing carcinoma	Ib1	2	0/21
IC16	Cervix	Squamous cell carcinoma papillary basaloid	Ib1	1	0/41
IC17	Cervix	Squamous cell carcinoma keratinizing	IIa1	3	0/43
IC18	Cervix	Squamous cell carcinoma keratinizing	Ia1	2	0/31
IC19	Cervix	Squamotransitional papillary carcinoma	Ib1	2	0/30
IC20	Cervix	Adenosquamous carcinoma	Ib1	2	0/40
IC22	Cervix	Squamous cell carcinoma keratinizing	Ib1	2	0/19
IC23	Cervix	Squamous cell carcinoma keratinizing	IIa1	2	0/60
IC24	Cervix	Squamous cell carcinoma keratinizing	IIa1	2	1/28
IC25	Cervix	Squamous cell carcinoma keratinizing	Ia2	2	0/42
IC27	Cervix	Squamous cell carcinoma keratinizing	Ib1	2	0/26

IE1	Uterus	Endometrioid adenocarcinoma	Ib	1	0/11
IE6	Uterus	Endometrioid adenocarcinoma	Ib	2	0/22
IE10	Uterus	Endometrioid adenocarcinoma	Ib	1	0/6
IE11	Uterus	Endometrioid adenocarcinoma	Ia	2	0/28
IE12	Uterus	Endometrioid adenocarcinoma	IIIc	2	3/25
IE13	Uterus	Adenocarcinoma endometrioid and mucinous	Ib	2	1/5
IE14	Ovary and uterus	Adenocarcinoma endometrioid with squamous metaplasia	Ib	2	0/29
IE15	Uterus	Endometrioid adenocarcinoma	0	0	0/23
IE16	Uterus	Clear cell adenocarcinoma	Ib	3	0/17
IE17	Uterus	Endometrioid adenocarcinoma	Ib	1	0/16
IE18	Uterus	Endometrioid adenocarcinoma	Ib	2	0/3
IE19	Uterus	Mucinous adenocarcinoma	Ib	2	0/16
IE20	Ovary and uterus	Endometrioid adenocarcinoma	Ib	1	0/12
IE23	Uterus	Endometrioid adenocarcinoma	Ia	1	0/4
IE24	Uterus	Malignant Müllerian mixed tumor	Ib	0	0/10
IE25	Uterus	Adenocarcinoma serous, papillary, and clear cell	Ib	3	0/56
IE27	Uterus	Endometrioid adenocarcinoma	IIIa	2	0/33
IE31	Uterus	Clear cell adenocarcinoma	IIIc	2	1/5

IV4	Vulva	Squamous cell carcinoma keratinizing	II	1	0/42
IV5	Vulva	Squamous cell carcinoma keratinizing	IIIb	3	3/35
IV6	Vulva	Squamous cell carcinoma keratinizing	IIIb	2	4/25
IV9	Vulva	Squamous cell carcinoma keratinizing	III	2	3/35
IV11	Vulva	Squamous cell carcinoma keratinizing	Ib	1	0/42
IV12	Vulva	Acrochordon verrucous	II	2	0/23
IV13	Vulva	Squamous cell carcinoma keratinizing	Ib	1	0/42
IV15	Vulva	Squamous cell carcinoma keratinizing	IIIb	1	1/30
IV16	Vulva	Squamous cell carcinoma keratinizing	IIIb	3	1/17

IC: cervical carcinoma, IE: endometrial carcinoma, IV: vulvar carcinoma. Diagnosis for patient IE15 was established following dilation and curettage for uterine polyps.

**Table 2:** Micrometastasis detection in 82 lymph nodes from the 46 patients employing both immunohistochemistry and real time quantitative RT-PCR (qRT-PCR) for CK19 and CA9.

Expression by immunohistochemistry	CK19 expression by qRT-PCR		CA9 expression by qRT-PCR	
	(+)	(−)	(+)	(−)
CK19				
(+) 11	11	0		
(−) 71	2	69		
CA9				
(+) 6			6	0
(−) 76			1	75
